# Optimising the caffeine nap for counteracting driver sleepiness in CPAP treated obstructive sleep apnoea patients

**DOI:** 10.1038/s41598-026-42894-1

**Published:** 2026-03-21

**Authors:** Ashleigh J. Filtness, Karl A. Miller, Sally Maynard, Adam Asmal, Thomas Kerwin, Andrew Hall, Anna Anund

**Affiliations:** 1 Transport Safety Research Centre, Loughborough University, Loughborough, UK; 2 School of Psychology, University of Nottingham, Nottingham, UK; 3 The Ohio State University, Columbus, USA; 4 The Hanning Sleep Laboratory, University Hospitals of Leicester NHS Trust, Leicester, UK; 5 NIHR Leicester Biomedical Research Centre & Leicester Diabetes Research Centre, University of Leicester, Leicester, UK; 6 Swedish National Road and Transport Research Institute (VTI), Linköping, Sweden

**Keywords:** Driver fatigue, Driver drowsiness, OSA, Countermeasure, Caffeine nap, Nappuccino, Sleep deprivation, Circadian rhythms and sleep, Wakefulness, Human behaviour, Sleep disorders

## Abstract

Driver sleepiness contributes to a substantial proportion of road crashes. Drivers experiencing sleepiness are advised to take a break and have a caffeinated drink followed by a short nap (caffeine nap). However, previous research advocating this countermeasure has not considered participants with obstructive sleep apnoea (OSA), the most prevalent sleep disorder. Across three studies the effectiveness of caffeine, nap opportunity and caffeine nap countermeasures on subjective sleepiness (KSS), objective sleepiness (Alpha and Theta activity) and driving performance (standard deviation of lateral position and out-of-lane events) are considered. Twenty-one CPAP treated OSA participants (mean age = 59 years) engaged with a protocol of six laboratory visits: one after a normal night’s CPAP-treated sleep and five after sleep restriction (4 h CPAP-treated sleep), driving a monotonous simulated scenario before and after a countermeasure. Results showed that two cans of coffee (255 mg caffeine) mitigated driver sleepiness more than one can (127.5 mg) and little benefit to 30 min compared with 15 min nap opportunity. An optimised caffeine nap of two coffees followed by a 15 min nap opportunity provides some temporary benefit, but for OSA drivers a caffeine nap offers little practical improvement compared to two coffees alone. All countermeasures are temporary and cannot replace a good night of sleep before driving.

## Introduction

Obstructive Sleep Apnoea (OSA) is considered to be the most prevalent sleep disorder, with an estimated global prevalence of over one billion people^1^. It is characterized by repeated collapse of the airway at night, with sufferers experiencing interrupted sleep impacting their health and daytime alertness. The prevalence of OSA is increasing as improved diagnosis coincides with a population-level increase in obesity^2^. Effective treatment with Continuous Positive Air Pressure (CPAP) is widely available in developed nations, and is the primary treatment provided by the UK National Health Service (NHS)^3^. Although untreated OSA is a road safety concern with a mean crash-rate ratio within 1.21–4.89^4^, once treated many OSA patients are fit to drive if excessive sleepiness is resolved; post-treatment, the incidence of road traffic collisions for this group can be substantially reduced^5^^,^^6^ and similar to that of healthy controls^7^.

The importance of OSA treatment to maintain road safety is widely acknowledged, with many countries having associated driver licencing requirements^8^ (UK regulations—location of this data collection). A diagnosis of mild, moderate or severe OSA without excessive sleepiness means a person may continue to drive as normal and does not need to notify the Driver Vehicle Licencing Authority (DVLA), as they are not considered to be a road safety risk. The UK Government website defines excessive sleepiness as having had difficulty concentrating and finding yourself falling asleep whilst driving. The requirement is to inform the DVLA due to having confirmed “moderate or severe obstructive sleep apnoea syndrome (OSAS), with excessive sleepiness” and refrain from driving^9^. This means that there are many diagnosed and treated OSA drivers on UK roads, as following treatment they may drive if they do not have excessive sleepiness. While this is not a road safety concern under normal sleep circumstances, our previous research has demonstrated that drivers with OSA can have a greater vulnerability to sleep loss even with the appropriate treatment^10^ and impact safe driving^11^.

Driver sleepiness is a common experience with 65% of drivers having felt sleepy while driving in the last 5 years^12^ and 20% of European car drivers reporting that they have driven at least once in the last month while so sleepy that they had trouble keeping their eyes open^13^. In the USA it has been estimated that around 1.5% of the total distance driven is completed while fatigued^14^. In general people are able to recognise their own sleepiness^15^ and therefore have the opportunity to act. While the safest act would be to cease driving, for many the motivation to complete a journey is high and temporary countermeasures are sought^16^. Commonly reported countermeasures cited by drivers include stopping for a walk, opening a window, turning up music, having caffeine and napping^17^. OSA drivers report similar actions but are more likely to nap^18^^,^^19^ and use multiple measures^18^ than those without OSA. Regardless of popularity the only countermeasures with notable scientific evidence supporting their efficacy for increasing alertness are: caffeine^20,21^, napping^23^ and a caffeinated nap^23^^,^^24^^,^^25^^,^^26^^,^^27^. Overall, the caffeine nap has demonstrated the strongest evidence of benefit in situations of comparison to either element alone. The caffeine nap (also sometimes referred to as a nappuccino) has been adopted within road safety, for example, it is recognised by the NHTSA^28^ of the USA, which recommends consuming caffeine prior to a 20-min nap to increase alertness for a short period of time. Despite the universality of this advice, the evidence-base is informed by research with “healthy” individuals and has been included in the UK Driver and Vehicle Standards Agency (DVSA) *Essential Guide to Driving*^29^. However, there is no empirical evidence for effectiveness in treated OSA drivers.

The current work seeks to identify the optimal composition for a caffeinated nap to support driving safety with treated OSA drivers following sleep restriction to 4 h (delayed bedtime) with CPAP treatment. Safety was evaluated using a driving simulator. To replicate a real-world setting caffeine was provided using commercially available drinks which are widely on sale in the UK market, with no control for body size. Nap opportunity was limited in location to within the driver’s seat of a passenger car—in line with the rest facility many drivers have access to while taking a break during a journey. The following research questions were addressed:

- Study 1: Does 255 mg of caffeine provide greater benefit than 127.5 mg of caffeine, to mitigate driver sleepiness in CPAP treated OSA drivers following sleep restriction to 4 h?
- Study 2: Does a 30 min nap opportunity provide greater benefit than a 15 min nap opportunity, to mitigate driver sleepiness in CPAP treated OSA drivers following sleep restriction to 4 h?
- Study 3: Can an optimised caffeine nap (made up of the most effective individual caffeine and nap components) mitigate driver sleepiness in CPAP treated OSA drivers following sleep restriction to 4 h, compared to driving before the caffeine nap and driving when alert (following a normal night of sleep)?

## Results

### Participants

21 participants (6 female, 15 male) aged between 33 and 83 years (M = 59, SD = 11.5) took part. All held a full UK driver’s licence and had been driving for between 14 and 60 years (M = 41.1, SD = 11.8), with a self-reported annual mileage ranging from 500 to 30,000 (M = 7119.0, SD = 5835.0). All participants reported habitually consuming no more than 5 cups of coffee per day, with the majority (81%) consuming 3 or fewer cups of coffee per day. Two participants dropped out part way through, resulting in 21 participants completing the two lab visits for Study 1 trialling caffeine strength (127.5 mg (C1) vs. 255 mg (C2)) and the two lab visits for Study 2 trialling nap opportunity (15 min nap (N15) vs. 30 min nap (N30)). Nineteen participants completed the additional two lab visits for Study 3, experiencing three different alertness statuses: once following a normal night of sleep (alert) and following sleep restriction where they drove once before and once after a caffeine nap (CN). The two participants who did not complete the final two CN visits were not noticeably different from the 19 that completed the full protocol.

All participants had been diagnosed with OSA for minimum of 6 months and maximum of 31 years (M = 12.0 SD = 9.4) and were being treated with CPAP.

### Sleep restriction manipulation check

Total sleep time (TST) was significantly varied on the nights before the six laboratory visits [*F (1.59,28.70)* = 108.544, *p* < 0.01, η_p_^2^ = 0.858]. Post hoc tests confirmed TST to be significantly longer prior to visit 1 (alert) than all of the five sleep restriction conditions for which participants had been instructed to sleep for 4 h (240 min) by delaying bedtime, with no difference between the five sleep restriction conditions (*p* < *0.05)*, see Table 1.

**Table 1:** Total sleep time prior to each lab visit: normal sleep duration(alert), and 4 h of sleep restriction for each countermeasure evaluation (1 can of coffee (C1)), 2 cans of coffee (C2), 15-min nap opportunity (N15), 30-min nap opportunity (N30), and caffeine nap (2 cans of coffee plus a 15 min-nap opportunity CN)).

	Alert	C1	C2	N15	N30	CN
Number of participants	21	21	21	21	21	19
TST minutes mean (Standard deviation)	430.05 (82.77)	229.38 (17.78)	224.29 (30.35)	222.48 (21.48)	218.71 (21.81)	216.79 (17.04)
Range	235–595	203–266	124–262	184–255	171–265	186–248

Prior to all lab visits participants were instructed to use their CPAP machine as usual. There was no difference in Apnoea-Hypopnea Index (AHI) across the six nights [X^2^(5) = 5.032, *p* = *0.412]*, and no difference between number of minutes of CPAP use between the five restricted nights of sleep [X^2^(4) = 1.686, *p* = *0.793],* see Table 2.

**Table 2:** CPAP metrics per lab visit: normal sleep duration(alert), and 4 h of sleep (1 coffee intervention (C1)), 2 cans of coffee intervention (C2), 15-min nap opportunity (N15), 30-min nap opportunity (N30), and Caffeine nap (2 cans of coffee plus.

	Alert	C1	C2	N15	N30	CN
Number of participants	20 (AHI = 19)	19	20	20	21 (AHI = 20)	19
CPAP use minutes mean (standard deviation)	470.00 (75.27)	252.05 (26.27)	245.15 (19.67)	255.60 (53.85)	250.52 (38.32)	260.26 (36.49)
Range of CPAP use minutes	258–619	223–236	209–312	167–435	188–388	224–354
AHI Mean (standard deviation)	2.18 (2.18)	1.75 (1.69)	2.20 (2.95)	2.04 (2.21)	1.35 (1.00)	1.55 (1.53)
AHI range	0.2–8.7	0–5.7	0–11.9	0–7.7	0.4–3.8	0–5.6

## Study 1: Caffeine dose

### Driving metrics

#### Standard deviation of lateral position (SDLP)

The LMM for SDLP converged successfully and provided an adequate fit to the data (AIC =  −  521.98; BIC = − 515.46). Higher caffeine strength did not have a significant impact on standard deviation of lateral position [*F*(1,173.69) = 0.40, *p* = 0.53]. There was a significant main effect of drive duration [*F*(4, 173.14) = 6.31, *p* < 0.001]. Pairwise comparison identified 0–15 min to be significantly lower than 16–30 min (*p* = 0.003), 46–60 min (*p* = 0.02), and 61-75 min (*p* < 0.001), while 31-45 min was also smaller than 60–75 min (*p* = 0.04) suggesting that variability in lane position started early on in the drive and increased as time passed. There was no interaction [*F*(4, 173.14) = 1.60, *p* = 0.18].

#### Minor out of lane events

The overall model fit for the negative binomial regression was acceptable (QIC = 334.85, QICC = 330.57). There was no effect of caffeine strength on minor out of lane events [Wald X^2^(1) = 2.81, *p* = 0.09]. There was an effect of driving duration [Wald X^2^(4) = 47.06, *p* < 0.001] evidencing minor out of lane events increase over time. Pairwise comparisons revealed fewer minor out of lane events in 0-15 min compared to 16–30 min (*p* < 0.001), and 61–75 min (*p* = 0.04). There were also more minor out of lane events in the 15 min directly prior to the intervention (16–30 min) compared to the 15 min following the intervention (31–45 min) (*p* = 0.001) suggesting benefit of caffeine, and more minor out of lane events at the end of the drive (61–75 min) compared to the 15 min immediately after the intervention (31–45 min) (*p* = 0.048).

**Fig. 1:**
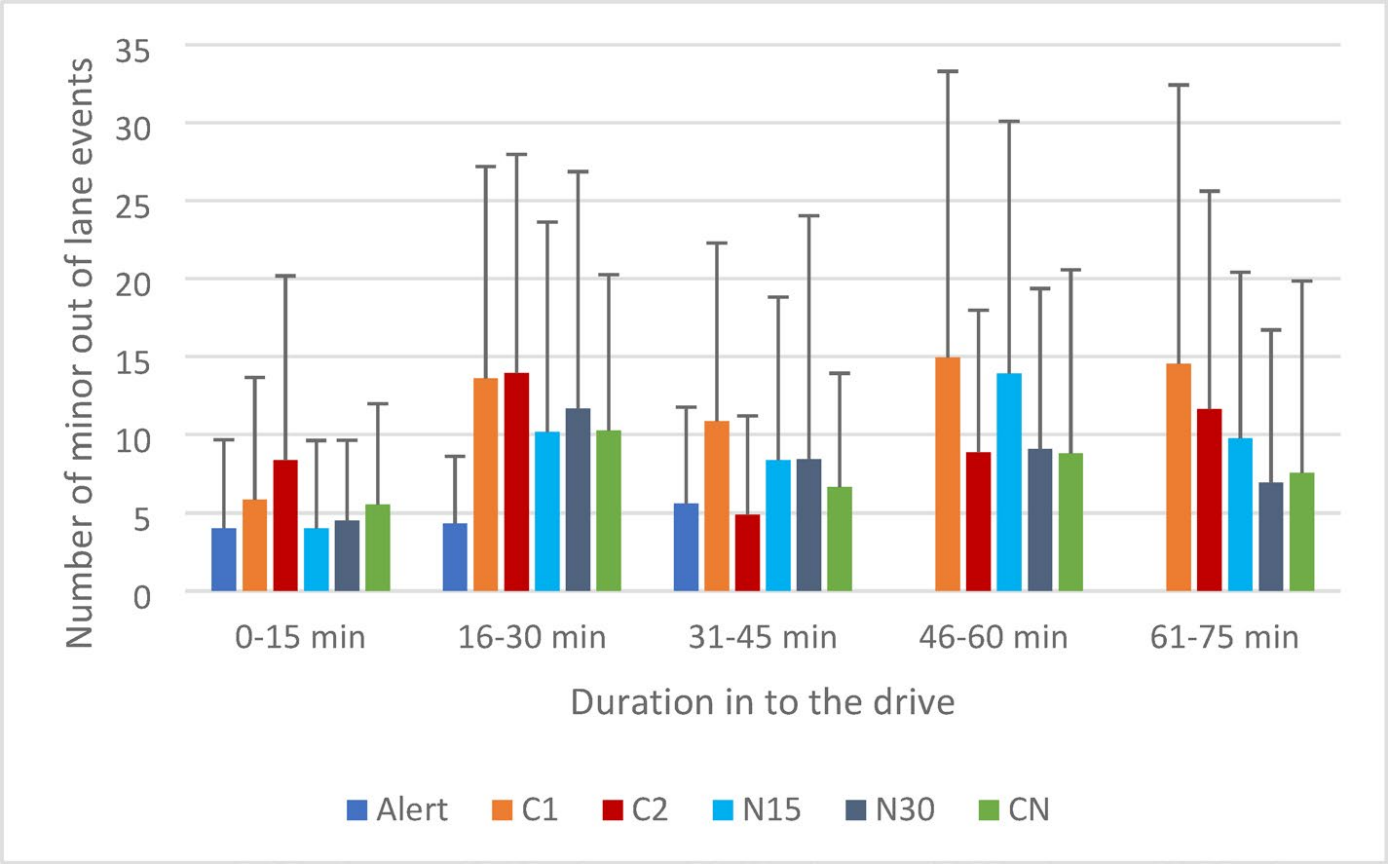
Mean number of minor out of lane events (two wheels touch a lane demarcation line) per 15 min of driving. Error bars represent standard deviation.

There was a significant interaction [Wald X^2^(4) = 32.79, *p* < 0.001] such that there were more minor out of lane events in the 15 min prior to the intervention compared to the 15 min following the intervention for C2 (*p* = 0.01) but not for C1 (*p* = 1.00), indicating C2 reduced minor out of lane events whereas C1 did not. Minor out of lane events are shown in Fig. 1.

#### Major out of lane events

**Fig. 2:**
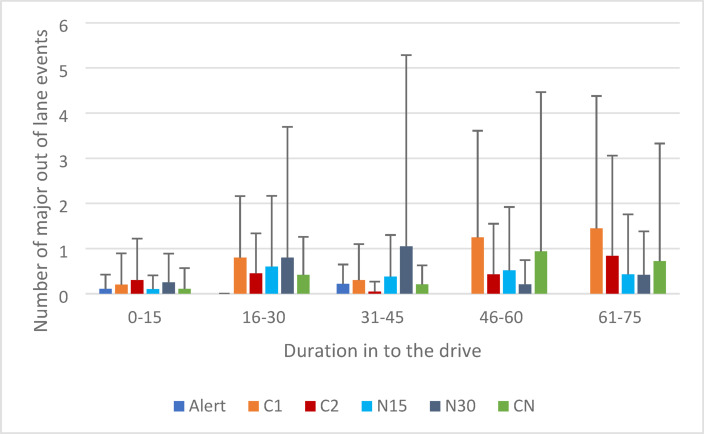
Mean number of major out of lane events (all four wheels touch or cross a lane demarcation line) per 15 min of driving. Error bars represent standard deviation.

The model provided an adequate fit to the data (QIC = 249.95, QICC = 233.89). There was no effect of caffeine strength on major out of lane events [Wald X^2^(1) = 1.83, *p* = 0.18]. There was an effect of driving duration [Wald X^2^(4) = 17.07, *p* = 0.002]. Pairwise comparisons revealed the number of major out of lane events increased over time, with a higher number of major out of lane events in the 15 min prior to the intervention (16–30 min) compared to the 15 min following the intervention (31–45 min) (*p* = 0.004). There was no significant interaction [Wald X^2^(4) = 7.10, *p* = 0.13]. Indicating that both C1 and C2 reduced major incidents. Major out of lane events are shown in Fig. 2.

### Subjective sleepiness

**Fig. 3:**
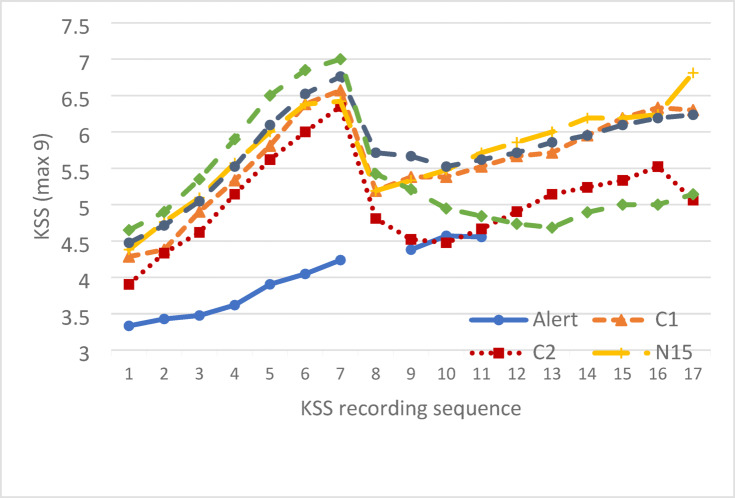
Mean KSS scores taken every 5 min of driving. An additional KSS was taken after countermeasure exposure prior to resuming driving in all intervention conditions, but driving was not paused in the alert condition (hence break in the line).

The model converged successfully (AIC = 666.46, BIC = 673.05). There was a main effect of caffeine strength such that KSS in the C2 condition was significantly lower than for C1 [*F*(1,180.03) = 16.03, *p* < 0.001]. The mean change in KSS for the 15 min of driving immediately before and after the caffeine was a reduction of 0.885 in C1 and 1.3651in C2. There was also a significant main effect of driving duration [*F*(4,180.03) = 15.97, *p* < 0.001]. Pairwise comparisons showed that KSS during 0–15 min was lower than 16–30 min (*p* < 0.001) and 61–75 min (*p* < 0.001). KSS during 16–30 min was significantly higher than 31–45 min (*p* < 0.001), and 46–60 min (*p* = 0.02). Indicating that the caffeine made participants feel more alert for up to an hour after consumption. Finally, during 61–75 min KSS was significantly higher than 31–45 min (*p* < 0.001). There was no significant condition by duration interaction [*F*(4,180.03) = 0.85, *p* = 0.50]. Mean KSS scores are displayed in Fig. 3.

After the two drives the participants were asked if the countermeasure made them feel more awake, 52% stated that C1 did and 80% stated that C2 did. Using a 7-point scale participants rated how effective they felt the countermeasure to be, and how likely they were to use it in real life. C2 was considered significantly more effective than C1 [Z = − 2.309, *p* = *0.021*], but there was no difference in likelihood to be used [Z = − 1.933, *p* = *0.053].*

### Objective sleepiness

The model converged successfully (AIC = 1247.76, BIC = 1253.91). The effect of caffeine strength on standardised combined alpha and theta activity was not significant [*F*(1,156.76) = 0.06, *p* = 0.82]. There was no effect of drive duration [*F*(4,142.73) = 0.41, *p* = 0.80]. The interaction between condition and drive duration was also not significant [*F*(4,142.73) = 0.46, *p* = 0.76].

## Study 2: Nap

### Driving metrics

#### Standard deviation of lateral position

The model converged successfully (AIC = − 534.29, BIC = − 527.79). There was no significant effect of the duration of nap duration [*F*(1, 171.48) = 2.38, *p* = 0.13]. There was a significant effect of drive duration [*F*(4, 170.99) = 7.43, *p* < 0.001]. Pairwise comparisons once again showed lower variations at 0–15 min compared to all other time points (all *p*’s < 0.001, excluding comparison to 31–45 min *p* = 0.03). There was no interaction [*F*(4, 170.99) = 1.04, *p* = 0.39].

#### Minor out of lane events

The model provided an adequate fit to the data (QIC = 365.10, QICC = 355.74). There was no effect of nap duration [Wald X^2^(1) = 0.56, *p* = 0.46]. There was a significant effect of driving duration [Wald X^2^(4) = 165.08, *p* < 0.001]. Pairwise comparisons revealed fewer minor out of lane events during 0–15 min compared to 16–30 min (*p* = 0.01) and 46–60 min (*p* = 0.003). There were also fewer out of lane events during 46–60 min compared to 61–75 min (*p* = 0.048).

There was also a significant interaction [Wald X^2^(4) = 11.46, *p* = 0.02], pairwise comparisons indicated that, within N15 departures were significantly higher at 40–60 min than at 0–15 min (*p* = 0.01), however there was no comparable increase was for N30. Minor out of lane events are shown in Fig. 1.

#### Major out of lane events

The model provided an adequate fit to the data (QIC = 272.20, QICC = 238.04). There was no effect of nap duration [Wald X^2^(1) = 0.55, *p* = 0.46]. There was an effect of driving duration [Wald X^2^(4) = 28.41, *p* < 0.001], however no pairwise comparisons were significant. There was no interaction [Wald X^2^(4) = 7.16, *p* = 0.13]. Major out of lane events are shown in Fig. 2.

### Subjective sleepiness

The overall model fit was adequate (AIC = 584.06, BIC = 590.65). The effect of duration of nap opportunity was not significant, evidencing no benefit of N30 compared to N15 [*F*(1,180) = 0.01, *p* = 0.95].The mean change in KSS for the 15 min of driving immediately before and after the intervention was 0.881 in N15 and 0.8293 in N30. There was a significant effect of drive duration [*F*(4,180) = 22.48, *p* < 0.001]. Pairwise comparisons identified that KSS scores at 0–15 min were lower than all other time points (all *p*s < 0.01). KSS at 31–45 min was significantly lower than 16–30 min (*p* < 0.001), and 61–75 min (*p* < 0.001). Indicating that having a nap decreases subjective sleepiness but this increases again with subsequent driving. The condition by duration interaction was not significant [*F*(4,180) = 0.48, *p* = 0.75]. Mean KSS scores are displayed in Fig. 1.

After the two drives the participants were asked if the countermeasure made them feel more awake, 57% stated that N15 did and 71% stated that N30 did. Using a 7-point scale participants rated how effective they felt the countermeasure to be, and how likely they were to use it in real life. There was no significant difference in rating of effectiveness of N15 compared with N30 [Z = − 1.089, *p* = *0.276],* nor in likelihood to be used [Z = − 1.291, *p* = *0.197].*

### Objective sleepiness

The initial LMM failed to converge, even after simplifying the random-effects structure, using maximum likelihood estimation, and increasing iteration limits. Because convergence was not achieved, the parameter estimates are considered unreliable and are not interpreted further. To explore the data further, a simpler repeated-measures ANOVA was conducted. The effect of duration of nap opportunity was significant [*F*(1,13) = 0.5.460, *p* = 0.036, η_p_^2^ = 0.296]. There was no significant main effect of drive duration [*F*(4,52) = 0.367, *p* = 0.831, η_p_^2^ = 0.027]. The interaction between condition and drive duration was also not significant [*F*(4,52) = 0.546, *p* = 0.703, η_p_^2^ = 0.040], suggesting that objective sleepiness levels differed between the two testing days, but not because of the nap duration. When asked if they had fallen asleep, 62% and 80% of participants reported that they had, with 5% and 15% being unsure, during N15 and N30 respectively.

## Study 3: Caffeinated nap

Overall, two cans of coffee showed greater benefits compared with one can of coffee. However, there was limited additional benefit to a 30 min nap opportunity compared with a 15 min nap opportunity. Therefore, it was decided that the caffeine nap should consist of two cans of coffee and a 15 min nap opportunity. A 15 min nap was used due to the minimal difference between a 15 min and 30 min nap, and because this is the current recommendation for drivers without sleep apnoea.

### Driving metrics

#### Standard deviation of lateral position

The model converged successfully (AIC = − 260.79, BIC = − 255.40). There was no significant effect of alertness status [*F*(2,91.94) = 0.20, *p* = 0.82]. There was a significant effect of driving duration [*F*(1,90.05) = 4.66, *p* = 0.03] such that standard deviation of lateral position was higher during 16-30 min compared to 0–15 min. The interaction between alertness status by drive duration was also not significant [*F*(2,90.05) = 1.27, *p* = 0.29]. In exploratory analysis the variability of lateral position was significantly more in the 15 min leading up to a caffeine nap (0.38 m) than the 15 min following the caffeine nap (0.35 m) [t(15) = 2.320, *p* = 0.035]. SDLP (standard deviation of lateral position) did not differ between C2 and CN [t(15) = 0.477, *p* = 0.640].

#### Minor out of lane events

The model provided an adequate fit to the data (QIC = 178.14, QICC = 176.31). There was no overall effect of alertness on the number of minor out of lane events [Wald X^2^(2) = 1.95, *p* = 0.38]. There was a significant effect of driving duration [Wald X^2^(1) = 4.51, *p* = 0.03], such that there were more events during 16–30 min compared to 0–15 min. There was no significant interaction [Wald X^2^(1) = 4.16, *p* = 0.13].

Exploratory analysis identified no significant difference prior to the CN 16-30 min (mdn = 8, m = 10.26) compared to immediately after (mdn = 6.68, m = 5.00) a *CN* [Z = − 1.291, *p* = *0.197]*. There was no significant difference in minor out of lane events during 31-45 min for CN (mean 6.68, SD = 7.25) compared with C2 (m = 4.90, SD = 6.29) [Z = − 0.825, *p* = *0.409]*. Minor out of lane events are shown in Fig. 1.

#### Major out of lane events

The model provided an adequate fit to the data (QIC = 112.61, QICC = 100.74). There was no overall effect of alertness on the number of major out of lane events [Wald X^2^(2) = 1.36, *p* = 0.51]. There was no effect of driving duration [Wald X^2^(1) = 3.37, *p* = 0.07], and no significant interaction [Wald X^2^(1) = 0.01, *p* = 0.94].

In exploratory analysis there was no significant difference in major out of lane events prior to the CN during 16–30 min (mdn = 0, m = 0.42) compared with after the CN 31–45 min (mdn = 0, m = 0.21) [Z = − 0.877, *p* = *0.380]* 31–45 min for CN (m = 0.21, SD = 0.419). Nor was there a significant difference between after a CN compared with after C2 (m = 0.05, SD = 0.218) [Z = − 1.342, *p* = *0.180]*. Major out of lane events are shown in Fig. 2.

### Subjective sleepiness

The model converged successfully (AIC = 463.66, BIC = 469.17). There was a significant main effect of alertness status [*F*(2,97.51) = 5.72, *p* = 0.004]. Pairwise comparisons showed that KSS was lower during the alert drive compared to the time prior to the caffeine nap intervention (*p* = 0.004), however there was no difference between pre and post intervention (*p* = 0.67). There was a significant main effect of driving duration [*F*(1,96.41) = 14.41, *p* < 0.001] evidencing sleepiness to increase over time. However, there was no interaction between alertness status and drive duration [*F*(2,96.41) = 0.45, *p* = 0.64] indicating sleepiness progression with time on tasks to be a similar pattern regardless of alertness status.

Exploratory analysis identified a non-significant trend for KSS to be higher (sleepier) in the 15 min following CN (m = 5.11, SD = 0.32) compared with following C2 (m = 4.74, SD = 0.30) [t(18) = − 1.994, *p* = 0.061]. Mean KSS scores are displayed in Fig. 1.

After the two drives the participants were asked if the countermeasure made them feel more awake, 95% stated that CN did. Using a 7-point scale participants rated how effective they felt the countermeasure to be, and how likely they were to use it in real life. CN was rated as more effective than all other countermeasures, compared with C1 [Z = − 2.804, *p* = *0.005], N15* [Z = − 2.780, *p* = *0.005], N30* [Z = − 3.494, *p* < *0.001]* apart from *C2* [Z = − 1.698, *p* = *0.090].*However, CN was not more likely to be used than any other countermeasure apart from N15 [Z = − 2.161, *p* = *0.031].*

### Objective sleepiness

The initial LMM failed to converge, even after simplifying the random-effects structure, using maximum likelihood estimation, and increasing iteration limits. Since convergence was not achieved, the parameter estimates are considered unreliable and are not interpreted further. To explore the data further, a simpler repeated-measures ANOVA was conducted.

There was no significant main effect of driving duration [*F*(1,12) = ,0.137 *p* = 0.307, η_p_^2^ = 0.087]. The interaction between alertness status by driving duration was also not significant [*F*(2,24) = 0.821, *p* = 0.452, η_p_^2^ = 0.064. In exploratory analysis combined alpha and theta power did not significantly differ in the 15 min prior compared to 15 min after CN [t(13) = − 0.29, *p* = 0.78]. Exploratory analysis comparing 31–45 min (the 15 min immediately after the countermeasure) between C2 and CN identified no significant difference in AT post CN (m = − 0.40, SD = 0.08) than post C2 (m = − 0.70, SD = 0.21) [t(8) = 0.300, *p* = 0.772]. When asked if they had fallen asleep, 53% of participants reported that they had, 32% were unsure.

## Discussion

Following systematic evaluation, it is recommended that if a person with treated OSA experiences sleepiness, the course of action with greatest evidence of temporary effectiveness is to stop driving and drink two cans of coffee (255 mg caffeine). This action is more positive in comparison to one can of coffee (127.5 mg caffeine) as it increases subjective alertness and improves driving performance (decreases out of lane events). One can of coffee does not appear to be enough to counteract sleep loss for OSA patients despite positive findings being reported for 80 mg^21^, 150 mg^22^ and 200 mg^20^ of caffeine in young healthy adults. It is possible that a higher concentration of caffeine is necessary to observe positive impacts in treated OSA participants, as drivers with OSA have been reported to habitually consume higher amounts of caffeine than non-OSA drivers^19^. High habitual caffeine use brings further concerns such as increased likelihood of poor sleep and poorer driving safety indicators^30^. However, although two cans of coffee were considered to be more effective, they were not rated as more likely to be implemented, suggesting that participants perceive a barrier to use of this countermeasure.

Having a longer nap opportunity did not show enhanced benefit, with no difference in driving performance (out of lane events) or subjective sleepiness for the N30 compared with N15. It is possible that there is less evidence of positive impact from napping than caffeine due to some sleep inertia. This is particularly concerning as OSA drivers are more likely to report napping as a preferred countermeasure than drivers without OSA^18^^,^^19^. Sleep inertia is a transient period of reduced alertness and impaired performance immediately after waking^31^. There is some evidence that this is more severe when waking from deep sleep^32^, a state which is more likely to be achieved with a longer nap opportunity. All participants slept with their CPAP machine at night; this was not available to them in the nap opportunities during this study. The nap opportunity provided represents an emergency situation whereby a person is caught out and experiences sleepiness while driving and does not have their CPAP machine with them. Our previous research has shown that missing just one night of CPAP treatment has a significant impact on driving^11^, so it is likely that the potential restorative impact of a nap is also reduced without the CPAP machine. As research with young healthy adults has reported the benefit of a 15 min nap^22^, it is likely that the OSA is a factor reducing the effectiveness in the current work. Future research should investigate whether a nap opportunity with CPAP is beneficial.

The benefits of a caffeine nap (CN) have been widely reported in healthy populations of drivers^23^^,^^24^ and shift workers^25^^,^^26^, however, the current work did not find substantial evidence of CN benefit for OSA drivers. There was limited evidence of benefit to driving performance or on subjective sleepiness experience. This finding is contradictory to previous research reporting a CN to be beneficial^23^^,^^24^^,^^25^^,^^26^^,^^27^. This suggests that official guidance containing the advice to use a CN (e.g. National Highway Traffic Safety Administration) may not be appropriate for OSA drivers. A greater number of significant findings were reported in Study 1 comparing C1 and C2, than in Study 2 comparing N15 and N30. In Study 1 pairwise comparison consistently evidenced benefits to driving and to subjective sleepiness after, compared with before, the intervention. Whereas pairwise comparisons in Study 2 identified subjective sleepiness to decrease during driving immediately following a nap but no such significant benefit in driving metrics. It is possible that for treated OSA drivers the nap component of the CN is not providing any benefit, and in fact may be introducing a disbenefit in comparison to caffeine alone. Exploratory analysis supports this assumption by identifying minimal difference between C2 and CN. As CN does not appear to provide benefit above C2, it is probable that for treated OSA drivers a recommendation for two cans of coffee as a temporary countermeasure to driver sleepiness would be appropriate. Future research should systematically compare CN and C2. Considering that OSA is the most prevalent sleep disorder^1^, providing appropriate advice will be of great benefit for millions of drivers.

The benefits (if any) of all trialled countermeasures were temporary. This supports previous findings with non-OSA drivers that caffeine and naps only offer temporary benefit and cannot return driving performance to the equivalent of alert driving^23^. However, it should be noted that in the current work benefits often dissipated after 15 min. Time on task plays a role in driver sleepiness as well as loss of sleep as participants felt sleepier and had greater impairments of driving as the drive progressed. Advice to OSA drivers should include consideration of how to minimise drive durations particularly following sleep loss.

As with all experimental studies the findings are limited by the sample size. However, the repeated measures design maximises statistical power, suggesting that any statistical differences are true findings. The study was designed to represent a real-world situation and so no control was imposed on participant demographics such as age, weight or gender, the only requirements were driving exposure and at least 6 months of CPAP treated OSA. Similarly, no experimental control was placed on the countermeasure dosage or the duration of time spent not driving during the intervention. Instead, fixed caffeine dosage and nap opportunity were used, replicating those which might be used in the real world. A driving simulator provides a safe environment to investigate factors which impair driving, however, it should be noted that simulator driving is not directly transferable to on-road driving. Subjective sleepiness and driving impairment are likely to be greater than those which would be observed on real roads^33^^,^^34^. It is unknown whether napping might differ in the familiar setting of someone’s own vehicle compared to the driving simulator. Lack of nap polysomnography limits sleep inertia conclusions. Additionally, this work does not consider the circumstances which lead a person to drive when sleepy or individual motivation factors around countermeasure use.

Overall, treated OSA patients are able to safely drive when alert. Following sleep restriction to 4 h, participants report feeling sleepier and exhibit driving impairment. If a caffeine countermeasure is to be used, then two cans of coffee are more effective than one; if a nap countermeasure is to be used there is likely no additional benefit of a 30 min nap opportunity compared to a 15 min nap opportunity. A caffeinated nap of two coffees followed by a 15 min nap opportunity provides temporary benefit to subjective alertness and driving impairment. However, this is likely not a practical improvement compared to two coffees alone. All countermeasures are temporary and cannot replace a good night of sleep prior to driving.

## Methods

Ethical approval for this work was granted by Loughborough University Human Ethics Sub- Committee, application number 18298. Research has been performed in accordance with Loughborough University ethics requirements and the Declaration of Helsinki. All participants gave full informed consent prior to taking part.

### Design

The research used a repeated measures design across three studies. The same participants completed all three studies; in total this equated to 6 lab visits and 11 driving simulator drives per participant.

Study 1 considered the research question: is there a difference in subjective and objective sleepiness and driving ability following 1 can of ice coffee (C1) compared to 2 cans of ice coffee (C2) for sleep restricted (4 h sleep) drivers with treated OSA? Participants completed 75 min of simulator driving, 30 min continuous driving before the intervention, then 45 min continuous driving after the intervention. In all studies the post-intervention drive is longer than the pre-intervention drive so that the impact of the intervention over a longer period of time could be observed. The independent variable (IV; intervention) was the amount of caffeine consumed: either one can (250 ml; 127.5 mg caffeine) of iced coffee or two cans (500 ml; 255 mg caffeine) of iced coffee.

Study 2 considered the research question: is there a difference in subjective and objective sleepiness and driving ability following having a 15-min nap (N15), or a 30-min nap (N30) opportunity in the car, for sleep restricted (4 h sleep) drivers with treated OSA? Participants completed 75 min of simulator driving, 30 min continuous driving before the intervention, then 45 min continuous driving after the intervention. The IV (intervention) was the duration of sleep opportunity, 15 min or 30 min.

Study 3 considered the research question: can a caffeinated nap (CN) be used to improve subjective and objective sleepiness and driving ability following sleep restriction compared to fully rested for drivers with treated OSA? The make-up of the CN was informed by the outcome of Study 1 and 2. Participants completed one drive (45 min) while alert, following a normal night of sleep. Two drives were completed following sleep restriction to 4 h, a pre-intervention drive (30 min) and a post-intervention drive (45 min). The intervention experienced was a CN made up of two cans of coffee followed by a 15 min nap opportunity. The IV was the sleepiness condition (alert, sleep deprived before caffeine nap, sleep deprived after caffeine nap).

The dependent variables in the study were: self-reported sleepiness through the Karolinska Sleepiness Scale (KSS), objective sleepiness as measured by EEG (combined Alpha and Theta activity) and driving metrics: average speed, SDLP and out of lane events. Lane drifting is the most common manifestation of sleepy driving; a car wheel touching or crossing the left rumble strip or touching the right-side lane line was classified as a driving incident.

Total sleep time (as measured by actigraphy and self-report sleep diaries) and CPAP use were also recorded to confirm compliance with sleep restriction. Several self-report scales were used within pre and post drive questionnaires: the pre-drive questionnaire documented participants’ demographics and driving experience. The post-drive questionnaire was concerned with participants’ views about the fidelity of the simulator, their experiences of sleepiness and their views about the particular countermeasure they had just experienced.

### Participant recruitment

Participant recruitment was supported by the Leicester Sleep Apnoea Patient Association who advertised the study to their members.

A total of 26 participants began the study with another four signing up but not managing to attend any visits due to conflicting schedules. The main reasons for withdrawal for those that started but did not complete were: sim sickness (two participants), major life events including illness (two participants) and an inability/unwillingness to restrict sleep and/or attend six lab sessions on specific occasions (one participant). A final total of 21 participants took part.

### Apparatus, materials, and stimuli

#### The driving simulator

Testing took place in the Transport Safety Research Centre’s driving simulator at Loughborough University. The simulator features a Ford KA car set inside a special environment made up of three large screens that form an open cube around the front, left and right sides of the car providing approximately 240° of visual angle. The screens use ultra-short throw projectors and rear-view and side mirrors are projected onto the screens in the relevant positions. A digital speedometer inside the vehicle (in the standard position) shows the vehicle’s speed. Sounds from the car and surrounding traffic are also included to enhance the reality of the simulation.

For the driving task, participants were asked to drive along a simulated motorway at the national speed limit of 70mph. Throughout the study participants were asked to drive in the leftmost lane of the motorway, except when entering and exiting the motorway. The driving task was created in SCANeR software and consisted of an 80 km (~ 50 miles) loop of a 3 lane UK motorway. To ensure the task was fatiguing, no traffic was present on the carriageway with the participant’s vehicle. The motorway included four rest areas 20 km (~ 12.5 miles) apart each having on/off slip roads and parking spaces which were used by participants during their interventions. Rumble strips were also included to audibly alert drivers if their vehicle crossed into the hard shoulder or central reservation. The road was designed to be monotonous so as to best reflect the conditions in which a driver might need to take a caffeine nap.

#### EEG

Polysomnography was recorded using an Embletta MPR with ST + Proxy at a sampling rate of 15 Hz. Two electroencephalogram (EEG) channels (C3-M2 (main channel); C4-M1 (back up channel)), two electrooculogram (EOG) channels (EOG left-M2; EOG right-M1), and a chin electromyogram (EMG) (2 EMG electrodes) were used. Single use cup electrodes were used alongside Ten20 conductive paste.

Impedance was measured using a Digimeter D175 impedance meter. Impedance for EEG and EOG was deemed acceptably low when below 7 kΩ, whereas EMG was acceptable below 20 kΩ.

#### Actigraphy and self-reported sleepiness

The night before each of the six visits to the lab, participants recorded their sleep using a MotionWatch actigraph (MotionWatch, CamNTech Ltd, Cambridge, UK) and by completing a sleep diary. This was primarily to ensure that participants were indeed sleep restricted during the study.

During each of the recorded drives, participants were asked to report their subjective levels of sleepiness every five minutes. The level of subjective sleepiness during each drive was assessed by using the Karolinska Sleepiness Scale [KSS]^35^. Participants indicated how sleepy they felt on average in the previous five minutes using a nine-point scale (1 = extremely alert to 9 = very sleepy, can’t stay awake). This was reported verbally and recorded during the drive, with participants being signalled to report their sleepiness level by an electronic beeper.

KSS is a widely used measure of sleepiness which has been validated against objective measures of sleepiness (EEG)^35^^,^^36^, blink duration, and driving performance measures^37^.

### Procedure

Participants were asked to visit the lab a total of six times. Before each visit, participants completed a sleep diary and wore an actiwatch to record how much sleep was obtained the night before each drive. Participants were also asked to avoid caffeine before each visit.

Visit 1 served as both a screening visit to test for signs of simulator sickness and to familiarise participants with the procedure, as well as an alert condition in which participants’ sleep was not restricted the night before. Visit 1 occurred at 10am for all participants to ensure that they were not subject to fatigue due to circadian lows in the afternoon.

During visit 1, participants were given detailed information about the study, and they provided informed consent to take part in the research. They were then presented with a simulator sickness questionnaire (SSQ)^38^ before the study to check for any signs of sickness before starting. They were then taken to the simulator to complete a short practice drive (~ 5 min) to check for any signs of simulator sickness. If the participant experienced no simulator sickness and was happy to continue with the study, they were taken out of the car and had the electrodes attached for polysomnography. Following the attachment of the electrodes, participants were taken back to the car to complete an alert drive lasting 45 min.

Visits 2–5 involved participants returning to the lab following a night of sleep restriction to 4 h. This was measured by actigraphy, the sleep diary, and by checking how long the CPAP device was used the night before the study. In these visits, participants first completed a 30-min pre-intervention drive in the simulator (using the same road as visit 1) before completing one of the four caffeine and nap interventions. The individual conditions were: drinking one can of iced coffee, drinking two cans of iced coffee, having a 15-min nap, or a 30-min nap opportunity in the car. This was followed by a 45-min post-intervention drive. The second drive commenced immediately following the intervention to replicate an approach that drivers might take in the real world. Consequently the duration of time spent not driving while the intervention took place varied between conditions.

The iced coffee comprised 250 ml cans containing approximately 128 mg of caffeine per can, nutritional values can be found in Table 3. This was chosen as it is a popular and widely available form of caffeine commonly consumed by drivers at service stations. An off the shelf caffeine product was used rather than controlled caffeine volume by body weight or personalised adaptation based on habitual caffeine consumption, to maximise relevance by evaluating an intervention which is available to drivers in the real world. This decision limits any conclusion on absolute caffeine impact on individual participants, but the repeated measures design means that any observed difference between one or two cans of coffee is not a result of variation in participant demographics. In addition, the use of a canned drink ensured a consistent amount of caffeine (compared to freshly brewed coffee) and allowed the drink to be consumed quickly. To further increase the realism of the intervention, the iced coffee was consumed in the car.

**Table 3:** Nutritional information for the commercially available canned coffee drink used.

Liquid per can	Approx. caffeine per can	Caffeine per 100 ml	Total caff	Sugar per 100 ml	Sugar (g)	Energy per 100 ml	Energy (kj)
250 ml	127.5 mg	51 mg	140.25	4.9	13.475	165	453.75

When napping in the car, participants were asked to sleep in the driver’s seat (based on our earlier survey of napping in the car^19^). Participants were asked to bring anything with them which they would typically use to help them nap in the car such as eye masks or ear plugs, but this was optional. Participants were asked if they had fallen asleep after the nap opportunity.

After the intervention, participants completed a second post-intervention 45-min drive. During both of these drives, participants reported their sleepiness via KSS, as well as having sleepiness recorded by EEG.

The order of these visits was counterbalanced using a blocked design such that participants always completed the caffeine conditions consecutively, and the nap conditions consecutively. This gave a total of four counterbalance orders.

During the final visit (visit 6) participants once again completed a 30-min pre-intervention drive before completing an intervention. The intervention for the final visit was a combined caffeine nap (CN) consisting of two cans (500 ml; 255 mg caffeine) of iced coffee immediately followed by a 15 min nap opportunity. The specifics of the caffeine nap were based on the highest performing intervention from study 1 and study 2. The intervention was once again followed by a 45-min post-intervention drive.

### Data pre-processing

#### Prior sleep

A manipulation check of the sleep restriction was conducted. When participants arrived in the lab a visual check of sleep diary and actigraphy data took place to confirm sleep restriction prior to taking part. Using MotionWare software total time in bed (min), estimated total sleep time (min) and sleep efficiency (%) were extracted from actigraphy data. In total 125 prior sleep occasions occurred, on 24 (19%) occasions actigraphy data was not available. This was due to participants failing to wear the watch (n = 5) or the technical failure of the watch recording despite being used accurately by the participant (n = 3). Changes in the date of attendance at the lab were also a reason for the failure of data collection from the watch (n = 9). At the beginning of the study the software for reading the data from the watch was inaccessible to the team; this affected a total of three participants (across seven visits). On these 24 occasions the time in bed and total sleep time were estimated using the sleep diary.

CPAP use (number of minutes the machine was in use) and the number of breathing events recorded per hour (AHI) were recorded through observation of either the physical CPAP machine or the associated app e.g. myAir™. In total 125 prior sleep occasions occurred, on 5 (4%) occasions. For one participant prior to the Alert condition the number of minutes used was recorded but not the number of events.

#### KSS

KSS data was available for all drives completed in the simulator. One participant fell asleep during N30, the remainder of this drive was scored as KSS 9. For the purpose of statistical analysis 15 min epochs are used.

#### EEG

Of 124 drives completed in the simulator EEG data is available for 98 (79%). Missing data occurred because of excessive noise in the recording (n = 6), and technical failure of equipment (n = 20). Missing data resulted in the following complete sample sizes Study 1 n = 16, Study 2 n = 16 and Study 3 n = 15. This reflects 20 participants in the Alert condition, 19 in C1, 16 in C2, 18 in N15, 19 in N30 and 16 in CN. Upon subsequent review for outliers two extreme outliers were identified in the N30 and CN condition, resulting in Study 2 n = 14, Study 3 n = 13. On all occasions the channel C3-M2 is used apart from one visit for one participant where the back-up channel C4-M1 was analysed due to a disconnected electrode part way through in the primary channel. For the purpose of statistical analysis 15 min epochs are used.

EEG data for spectral analysis were extracted using RemLogic software in 4-s epochs. The data were subject to high and low bandpass filtering at 20 and 4 Hz to remove slow eye movements and muscle artefacts. For analysis, there was a focus on the combined Alpha-Theta powerband (4–11.99 Hz) which was exported for further processing.

Pre-processing and artefact removal occurred for data per participant. Data were segmented by individual simulator drives for each visit (pre and post intervention), manual inspection of the data removed any 4 s epoch which contained artefacts. On average artefact removal impacted 5.28% (SD = 4.57%) of EEG data. Data were then further divided into 15-min intervals. Within each interval any values which were more than 2 SD away from the mean were considered outliers and were replaced with the average of the preceding and following values. Finally, the data were standardised by computing Z-scores using the formula Z = (X − μ)/σ, where μ is the mean and σ is the standard deviation.

### Driving simulator

Of 124 drives completed in the simulator, simulator data is available for 117 visits (94.3%). Two of these drives were started but not completed as the (same) participant fell asleep part way through. Missing data occurred for some analysis from two files where the simulator recording cut out unexplainedly missing the final segment (61–75 min) even though the participant completed the drive, both during C2. Two files have not been included because of a failure in the simulator turning off and on again part way through, resulting in Study 1 n = 21, Study 2 n = 18, Study 3 n = 19. Upon subsequent review for outliers three extreme outliers for standard deviation of lateral position were identified and excluded in the CN condition, resulting in n = 16 for SDLP in Study 3. Outliers were not removed from out of lane events due to the lower frequency of this occurrence making outlier detection less reliable.

Driving simulation data was processed with Pydre^39^. Metrics were calculated in each of three time-blocks. Out of lane occurrences, which could be called lane deviations, are a commonly used safety performance indicator in driving simulator studies^15^. In the current work we distinguish between minor and major out of lane events. Minor events occur when two of the four vehicle tyres cross a lane demarcation line, indicating poor vehicle handling but to a level that might not intrude sufficiently into an adjoining lane to impact other traffic. A major out of lane event was defined as the driven vehicle crossing the lane line to an extent that a potential neighbouring vehicle would be forced out of their lane to avoid a collision, defined as being where the centre of the driven vehicle crosses the lane markings.. For a subsequent event to be counted the driven vehicle must return to the appropriate driven lane for at least 2 s in between events. This prevents counting as multiple events a situation where the driver is on the borderline of the lane markings.

### Statistical analysis

#### Prior sleep manipulation check

One-way ANOVA was used to compare total sleep time recorded by actigraphy between the six lab visits, Bonferroni correction was used for post-hoc paired comparisons. Kolmogorov–Smirnov test was used to identify data which were not normally distributed, as a result, total minutes of CPAP use and AHI were compared using a Friedman test rather than one-way ANOVA. For total minutes of CPAP use only the five lab visits following sleep restriction were analysed.

#### Study 1 and 2

Linear mixed-effects models (LMMs) were used to analyse subjective sleepiness (KSS), lane-keeping variability (SDLP), and EEG measures. Each model included two fixed within-subject factors: intervention type (2 levels: C1 and C2, or, N15 and N30) and driving duration (5 levels: 0–15 min, 15–30 min, 30–45 min, 45–60 min, 60–75 min). Participant number was included as a random intercept. Models were estimated using restricted maximum likelihood (REML) with Satterthwaite’s approximation for degrees of freedom. In cases where the model failed to converge, a simpler repeated measures ANOVA was used. Bonferroni-adjusted pairwise comparisons were used for post-hoc tests. Driving segments from 0 to 30 min occurred before the intervention, and 30–75 min occurred afterwards.

As both major and minor out of lane violations were count variables, they were analysed using negative binomial regression with a log link function. The model included intervention strength and driving duration as fixed within-subject factors, and participant as the clustering variable. Bonferroni-adjusted pairwise comparisons were used for post-hoc tests.

#### Study 3

Linear mixed-effects models (LMMs) were used to analyse subjective sleepiness (KSS), lane-keeping variability (SDLP), and EEG measures. Each model included two fixed within-subject factors: alertness status (3 levels: alert, sleepy before CN, sleepy after CN) and, (ii) duration of driving (2 levels: 0–15 min, 15–30 min). Participant number was included as a random intercept. Models were estimated using restricted maximum likelihood (REML) with Satterthwaite’s approximation for degrees of freedom. In cases where the model failed to converge, a simpler repeated measures ANOVA was used. Bonferroni-adjusted pairwise comparisons were used for post-hoc tests.

As with studies 1 and 2, major and minor out of lane violations were count variables,and were analysed using negative binomial regression with a log link function. The model included alertness status and driving duration as fixed factors, and participant as the clustering variable. Bonferroni-adjusted pairwise comparisons were used for post-hoc tests.

In situations where there was no significant main effect of alertness status, an exploratory 2-sided t test comparing before CN and after CN was completed to provide some understanding as to whether or not there was any impact of CN. Due to the impact of CN being less than expected from the literature, exploratory 2-sided t test comparisons between C2 and CN for 31-45 min (the 15 min immediately after the countermeasure) were completed. Exploratory results should be interpreted with caution due to the repetition of statistical testing increasing the chance of type 1 error. Kolmogorov–Smirnov test was used to identify data which were not normally distributed. All statistical analysis was completed in IBM SPSS 29. A significance level (α) of 0.005 was used.

## Data Availability

The datasets generated and analysed during the current study can be found in Loughborough University Research repository https://repository.lboro.ac.uk/. At the time of writing the dataset is embargoed until the funder has finalised the project, expected April 2026. To request data from this study outside of the repository please contact a.j.filtness@lboro.ac.uk.
